# Off-Label Medication Use in a Serbian Neonatal Intensive Care Unit: A Single-Center Retrospective Cohort Study

**DOI:** 10.3390/jcm15166183

**Published:** 2026-08-10

**Authors:** Nikola Martić, Jovana Jančić, Slobodan Spasojević, Nemanja Martić, Aleksandar Rašković, Marko Krstić, Milica Paut Kusturica

**Affiliations:** 1 Department of Pharmacology, Toxicology, and Clinical Pharmacology, Faculty of Medicine, University of Novi Sad, Hajduk Veljkova 3, 21000 Novi Sad, Serbia; 2 Faculty of Medicine, University of Novi Sad, Hajduk Veljkova 3, 21000 Novi Sad, Serbia; 3 Department of Neonatology, Institute for Child and Youth Healthcare of Vojvodina, Hajduk Veljkova 10, 21000 Novi Sad, Serbia; 4 Clinic for Nephrology and Clinical Immunology, University Clinical Centre of Vojvodina, Hajduk Veljkova 1, 21000 Novi Sad, Serbia; 5 Faculty of Chemistry, University of Belgrade, Studenski Trg 16, 11000 Belgrade, Serbia

**Keywords:** neonate, intensive care, neonatal, off-label use, drug therapy, unlicensed medications

## Abstract

**Background/Objectives:** The main objective of this study was to describe the prevalence and prescribing patterns of off-label and unlicensed medication use in a tertiary neonatal intensive care unit in Serbia and to evaluate differences according to gestational age. **Methods:** This retrospective single-center cohort study included 257 neonates hospitalized in a tertiary neonatal intensive care unit between January and December 2023. Medication prescriptions were classified as off-label or unlicensed according to the approved Summary of Product Characteristics in Serbia. Clinical and prescribing data were analyzed descriptively, with predefined patient-level outcomes compared across gestational-age groups using non-parametric analyses. **Results:** A total of 1919 distinct medication prescription records were identified, of which 1642/1919 (85.6%) were classified as off-label and 27/1919 (1.4%) as unlicensed. At the patient level, 252/257 neonates (98.1%) were exposed to at least one off-label medication, whereas 26/257 (10.1%) were exposed to at least one unlicensed medication. Anti-infectives for systemic use accounted for the largest therapeutic-class prescription count. Off-label prescribing was primarily related to age and dosing recommendations. Medication utilization varied across gestational age groups; however, extremely preterm neonates also had substantially longer hospitalizations, and the observed differences should therefore be interpreted as unadjusted prescribing patterns rather than an independent effect of gestational age. **Conclusions:** Off-label prescribing was highly prevalent in this Serbian tertiary neonatal intensive care unit, whereas patient exposure to unlicensed medications was relatively uncommon. The observed differences across gestational age groups should be interpreted in the context of differences in hospitalization duration and clinical complexity. These findings characterize prescribing patterns and regulatory gaps in neonatal pharmacotherapy and identify areas requiring further neonatal-specific clinical research.

## 1. Introduction

The pharmacological treatment of neonates is a complex issue, demanding substantial expertise in both neonatology and pharmacology. Key organ functions, including hepatic and renal metabolism and elimination, are still immature. Additionally, neonates exhibit distinct pharmacokinetic and pharmacodynamic profiles compared to older children and adults [1,2]. In the neonatal intensive care unit (NICU), this challenge is further heightened by severe illnesses, the extensive use of medications, and the combination of early gestational age with low birth weight [3]. Preterm neonates frequently depend on life support and experience significant organ immaturity. Neonates therefore represent a unique patient population in which pharmacotherapy requires careful consideration of developmental pharmacokinetics, pharmacodynamics, and clinical condition [4,5]. Moreover, data on the safety and efficacy of neonatal pharmacotherapy remain limited for many medications. As a result, up to 90% of neonates in NICU receive medications prescribed in an unlicensed or off-label (OL) manner [1,6].

OL use refers to the administration of a medication outside the conditions specified in the Summary of Product Characteristics (SmPC), including unapproved age group, indication, dosage, dosing frequency, or route of administration [7]. In contrast, unlicensed (UL) use applies to medications without marketing authorization, including modified formulations of previously approved medications or imported medications licensed in other countries [7,8]. This practice is neither illegal nor inappropriate and is supported by extensive clinical experience, often being acceptable or even recommended in clinical guidelines when no suitable alternative is available [8].

In recent years, with support from the global medical community, policies regarding medication prescriptions for pediatric populations have evolved, with a greater focus on this critical stage of life [9]. In this regard, the European Union introduced Regulation (EC) No. 1901/2006, which came into effect in 2007. The primary goal of this regulation was to collect data on the use of medications in pediatric populations across EU member states, identifying areas that require further research while minimizing the need for unnecessary clinical trials involving children or newborns [10,11]. A decade later, a report from the European Commission highlighted the positive impact of this regulation on the development of pediatric medicines, particularly in areas such as immunology-based therapies, antiviral medications, and treatments for congenital metabolic disorders. However, the regulation’s influence on the development of older OL medications has been limited. Although the emergence of new treatments may have reduced the urgency surrounding OL medication use, further research in this area remains valuable [8].

To date, no similar study has been conducted in the Republic of Serbia. Characterizing current neonatal prescribing practices may help identify regulatory and evidence gaps and provide baseline data for future clinical, regulatory, and multicenter research. Therefore, the aim of this study was to characterize off-label and unlicensed medication prescribing in the neonatal intensive care unit of the Institute for Child and Youth Healthcare of Vojvodina using a retrospective cohort design.

## 2. Materials and Methods

This retrospective study was conducted at the NICU of the Institute for Child and Youth Healthcare of Vojvodina in Novi Sad, Serbia. As the second-largest NICU in the country and the only level IIIb neonatal unit in the northern province of Serbia (Vojvodina), which serves a population of approximately two million, it represents a major regional center for neonatal care. The study covered a predefined one-year period from 1 January to 31 December 2023, providing a complete calendar year of admissions and medication exposure in the NICU. All neonates admitted during the study period were assessed for eligibility. Neonates who died within the first 24 h after admission and those transferred to another institution for further surgical treatment were excluded from the analysis. Neonates transferred within the Institute for continued neonatal care remained eligible for inclusion. Importantly, neonates who died after more than 24 h of hospitalization were retained in the study cohort and included in all analyses. The exclusion of neonates who died within the first 24 h was based on the study objective of characterizing medication utilization during ongoing NICU hospitalization, as very short hospital stays provide limited opportunity to assess prescribing patterns. This subgroup also represents a clinically distinct population with extremely severe underlying conditions and heterogeneous causes of early mortality, including conditions potentially unrelated to medication exposure, such as major congenital anomalies or profound perinatal compromise. This retrospective observational cohort study was conducted and reported in accordance with the Strengthening the Reporting of Observational Studies in Epidemiology (STROBE) statement [12].

Clinical and demographic data were retrieved from the hospital’s Health Information System and patient medical records, including sex, gestational age, Apgar score, birth weight, birth length, diagnoses, age at admission, length of hospitalization, pharmacological treatments, day of therapy initiation, duration of therapy, route of administration, dosing information, and any dose adjustments. All data were entered into Microsoft Excel 2016 (Microsoft Corp., Redmond, WA, USA) for organization and subsequent analysis. Certain categories of treatments were not included in the study. Specifically, intramuscular injections were excluded because this route of administration is not routinely used in our NICU. In addition, enteral nutrition products, suppositories, dietary supplements (e.g., vitamin D), and nasal drops were excluded because the study specifically focused on prescribed medicinal products used for the treatment of neonatal diseases and conditions. Inclusion of these products would have substantially increased the number of recorded interventions without contributing to the primary objective of evaluating off-label and unlicensed drug prescribing. Only pharmacological agents were evaluated. Off-label and unlicensed prescribing were classified according to the approved Summary of Product Characteristics (SmPC) authorized in the Republic of Serbia and available through the official database of the Medicines and Medical Devices Agency of Serbia (ALIMS) [13]. Off-label use was defined as prescribing outside the approved age, indication, dose, dosing interval, or route of administration. Unlicensed use referred to medications lacking formal marketing authorization or to compounded or reformulated products not covered by the approved marketing authorization. Medication preparation and administration followed the Institute’s standard operating procedures (SOPs). Medications requiring preparation or dilution were compounded in the central hospital pharmacy under aseptic conditions in a laminar airflow cabinet before being distributed to the NICU for administration.

Neonates were stratified into four gestational age (GA) groups for analysis: <28 weeks, ≥28 to <32 weeks, ≥32 to <37 weeks, and ≥37 weeks gestational age. Medication classifications were independently performed by a neonatologist and a clinical pharmacologist using the approved SmPC available through the ALIMS. Classifications were based on the approved indication, age, dose, dosing interval, route of administration, and formulation. No discrepancies in medication classification were identified because the evaluated medications represent routinely prescribed therapies in the study NICU and were assessed according to predefined regulatory criteria. Diagnosis codes for all hospitalized neonates were recorded according to the International Classification of Diseases (ICD-10), with each patient having at least one and, in some cases, multiple documented diagnoses. Diagnoses were summarized descriptively as part of the clinical characterization of the cohort but were not used for diagnosis-specific analyses of medication prescribing. The study was approved by the Ethics Committee of the Institute for Child and Youth Healthcare of Vojvodina under approval number 5828-2 in December 2023, following institutional and national ethical guidelines. As this was a retrospective study using anonymized data, informed consent was not required.

All statistical analyses were performed using SPSS Statistics version 21 (IBM Corp., Armonk, NY, USA). Descriptive statistics were used to summarize the study population and medication-utilization data. Continuous variables were summarized as mean ± standard deviation or median and interquartile range, as appropriate to the characteristics of the data, whereas categorical variables were summarized as frequencies and percentages. Comparisons across the four gestational-age groups were performed using the Kruskal–Wallis test for the following patient-level outcomes: length of hospitalization, number of distinct study-included medications per patient-day, and number of distinct off-label medications prescribed per neonate during hospitalization. The reported analyses represent global comparisons across the four gestational-age groups; no post hoc pairwise comparisons were performed. Effect size for Kruskal–Wallis analyses was expressed as epsilon-squared (ε^2^). Medication utilization according to Anatomical Therapeutic Chemical classes and patient exposure to individual off-label and unlicensed medications were analyzed descriptively; no inferential comparisons or significance markers were applied to these medication-specific analyses. For prescription-level analyses, each distinct medication prescribed to an individual neonate during hospitalization was counted once; repeated doses or repeated documentation of the same medication in the same patient were not counted as separate prescription records. Patient-level exposure was defined as the number of neonates who received at least one medication from the respective therapeutic class or regulatory category during hospitalization. A two-sided *p*-value < 0.05 was considered statistically significant.

## 3. Results

During the study period, 270 neonates were admitted to the neonatal intensive care unit and assessed for eligibility. Thirteen neonates were excluded from the analysis: eight died within the first 24 h after admission, and five were transferred to another institution in Belgrade for further surgical treatment. The final study cohort therefore comprised 257 neonates. Of these, 13 neonates died after more than 24 h of hospitalization and remained included in all analyses. The final cohort comprised 151 males (58.8%) and 106 females (41.2%). The majority were born at term (≥37 weeks gestational age, *n* = 154; 59.9%), followed by moderate to late preterm neonates (≥32 to <37 weeks, *n* = 50; 19.4%), very preterm neonates (≥28 to <32 weeks, *n* = 41; 16.0%), and extremely preterm neonates (<28 weeks, *n* = 12; 4.7%). The most frequently documented diagnoses were respiratory distress syndrome (P22.0; *n* = 113), mild and moderate perinatal asphyxia (P21.1; *n* = 79), low birth weight of 1000–2499 g (P07.1; *n* = 75), and severe perinatal asphyxia (P21.0; *n* = 41). The mean birth weight was 2589.6 ± 1028.9 g, and the mean birth length was 46.1 ± 6.5 cm. The median length of hospitalization for the overall cohort was 8 days (IQR 5–11) and differed across gestational-age groups (Kruskal–Wallis H = 59.31, *p* < 0.001, ε^2^ = 0.223). Median hospitalization was 32.5 days (IQR 17.8–50.3) in neonates born at <28 weeks, 12.0 days (IQR 8.0–20.0) at ≥28 to <32 weeks, 8.0 days (IQR 5.3–13.5) at ≥32 to <37 weeks, and 7.0 days (IQR 5.0–8.8) at ≥37 weeks. When medication exposure was descriptively standardized for hospitalization duration, the median number of distinct medications per patient-day was 0.45 (IQR 0.40–0.63), 0.58 (IQR 0.47–0.73), 0.86 (IQR 0.65–1.00), and 0.83 (IQR 0.60–1.14), respectively (Kruskal–Wallis H = 25.31, *p* < 0.001, ε^2^ = 0.088). The mean Apgar scores were 6.4 ± 2.7 at 1 min and 7.7 ± 2.0 at 5 min after birth (Table 1).

A total of 1919 distinct medication prescription records were identified during the study period. Of these, 1642/1919 (85.6%) were classified as off-label, 27/1919 (1.4%) as unlicensed, and 250/1919 (13.0%) as on-label. At the patient level, 252/257 neonates (98.1%) were exposed to at least one off-label medication, whereas 26/257 (10.1%) were exposed to at least one unlicensed medication during hospitalization. A total of 68 distinct medications were identified, of which 56 were classified as off-label, 4 as unlicensed, and 8 as on-label according to the approved Serbian SmPC.

Table 2 presents medication utilization according to the Anatomical Therapeutic Chemical classification system, stratified by gestational age. For each therapeutic class, prescription counts are reported separately from patient-level exposure, defined as the number and proportion of neonates who received at least one medication from the respective therapeutic class. A single neonate could contribute more than one prescription within the same therapeutic class if more than one distinct medication from that class was prescribed, whereas each neonate was counted only once when calculating patient exposure. Anti-infectives for systemic use accounted for the highest number of prescriptions (*n* = 916) and were administered to 250 of 257 neonates (97.3%). Nervous system medications accounted for 342 prescriptions and were administered to 198 of 257 neonates (77.0%), while cardiovascular medications accounted for 306 prescriptions and were administered to 150 of 257 neonates (58.4%). Descriptive differences in medication utilization were observed across gestational-age groups; however, these findings represent unadjusted patterns of prescribing and should not be interpreted as demonstrating an independent effect of gestational age on medication exposure.

At the patient level, 252/257 neonates (98.1%) were exposed to at least one off-label medication during hospitalization, whereas 26/257 neonates (10.1%) were exposed to at least one unlicensed medication. Unlicensed medication exposure was exclusively related to modified formulations, including povidone-iodine eye drops, propranolol, ursodeoxycholic acid, and captopril. Table 3 summarizes the most frequently prescribed off-label medications, the 24 less frequently used off-label medications grouped as “Other,” and all four unlicensed medications, whereas Table A1 presents the eight on-label medications included in the study and their regulatory classification according to the Serbian Summary of Product Characteristics. Among the off-label medications presented in Table 3, the highest patient exposure rates were observed for gentamicin (90.7%), ampicillin (65.4%), ceftazidime (54.9%), fentanyl (43.6%), and furosemide (41.2%). Descriptive differences in patient exposure to individual off-label medications were observed across gestational-age groups, with extremely preterm neonates showing higher exposure to several commonly prescribed medications, including gentamicin, ampicillin, ceftazidime, meropenem, fluconazole, and budesonide. These medication-specific comparisons are presented descriptively and should not be interpreted as statistically significant between-group differences. The most common reasons for off-label classification were discrepancies related to the approved age, dose or dosing interval, and indication.

**Figure 1:**
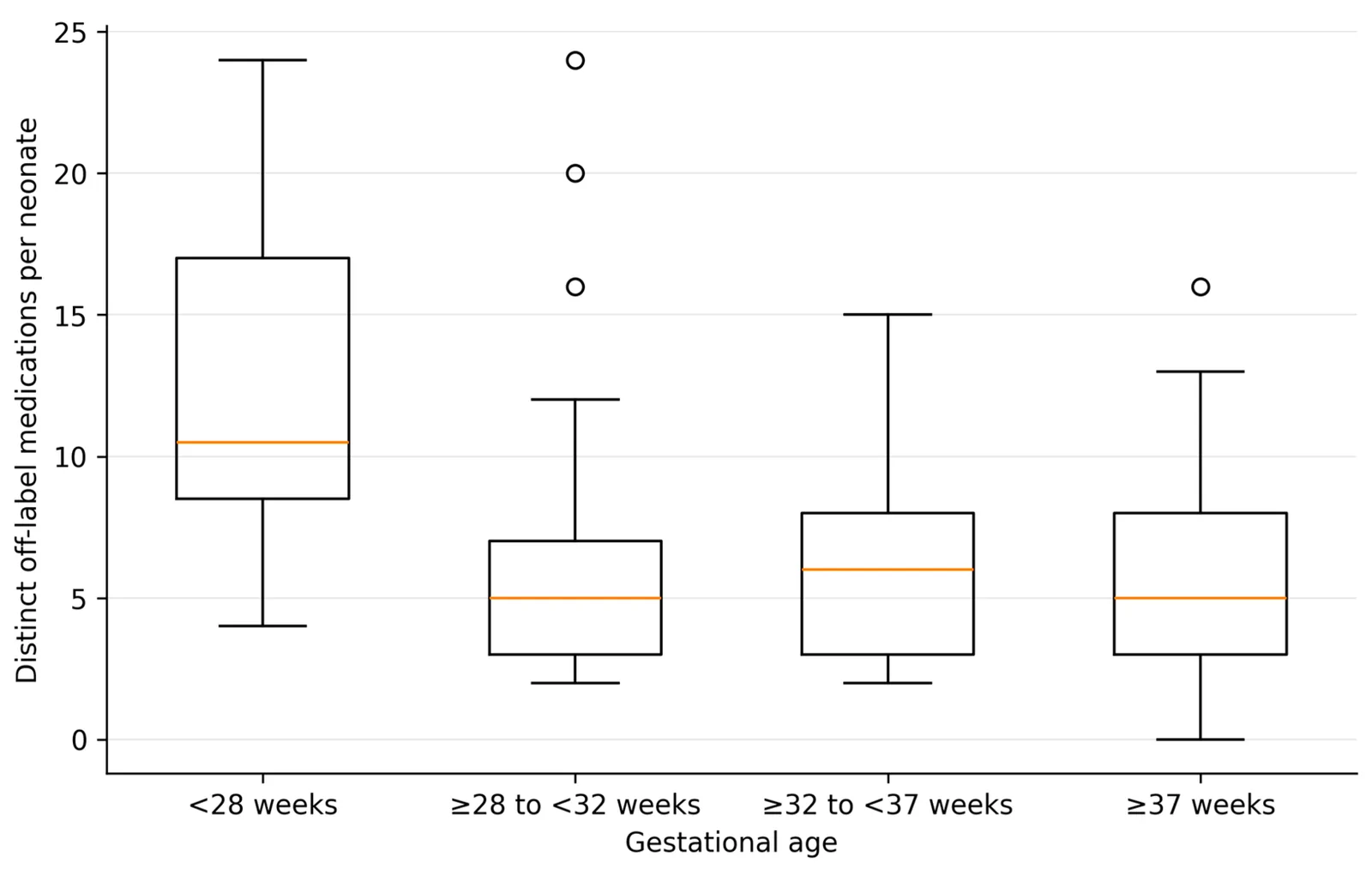
Distribution of the number of distinct off-label medications prescribed per neonate according to gestational age. Boxes represent the interquartile range, horizontal lines represent the median, and whiskers extend to the most extreme observations within 1.5 times the interquartile range; observations beyond the whiskers are displayed individually. An overall difference among gestational-age groups was assessed using the Kruskal–Wallis test (H = 18.29, *p* < 0.001, ε^2^ = 0.060).

The number of distinct off-label medications prescribed per neonate during hospitalization differed across gestational-age groups (Kruskal–Wallis H = 18.29, *p* < 0.001, ε^2^ = 0.060). Neonates born at <28 weeks had a median of 10.5 distinct off-label medications per patient (IQR 8.5–17.0), compared with 5.0 (IQR 3.0–7.0) in those born at ≥28 to <32 weeks, 6.0 (IQR 3.0–8.0) at ≥32 to <37 weeks, and 5.0 (IQR 3.0–8.0) at ≥37 weeks (Figure 1; Table A2). The global test indicates an overall difference among gestational-age groups; no individual between-group difference is inferred from this analysis without post hoc comparisons.

**Figure 2:**
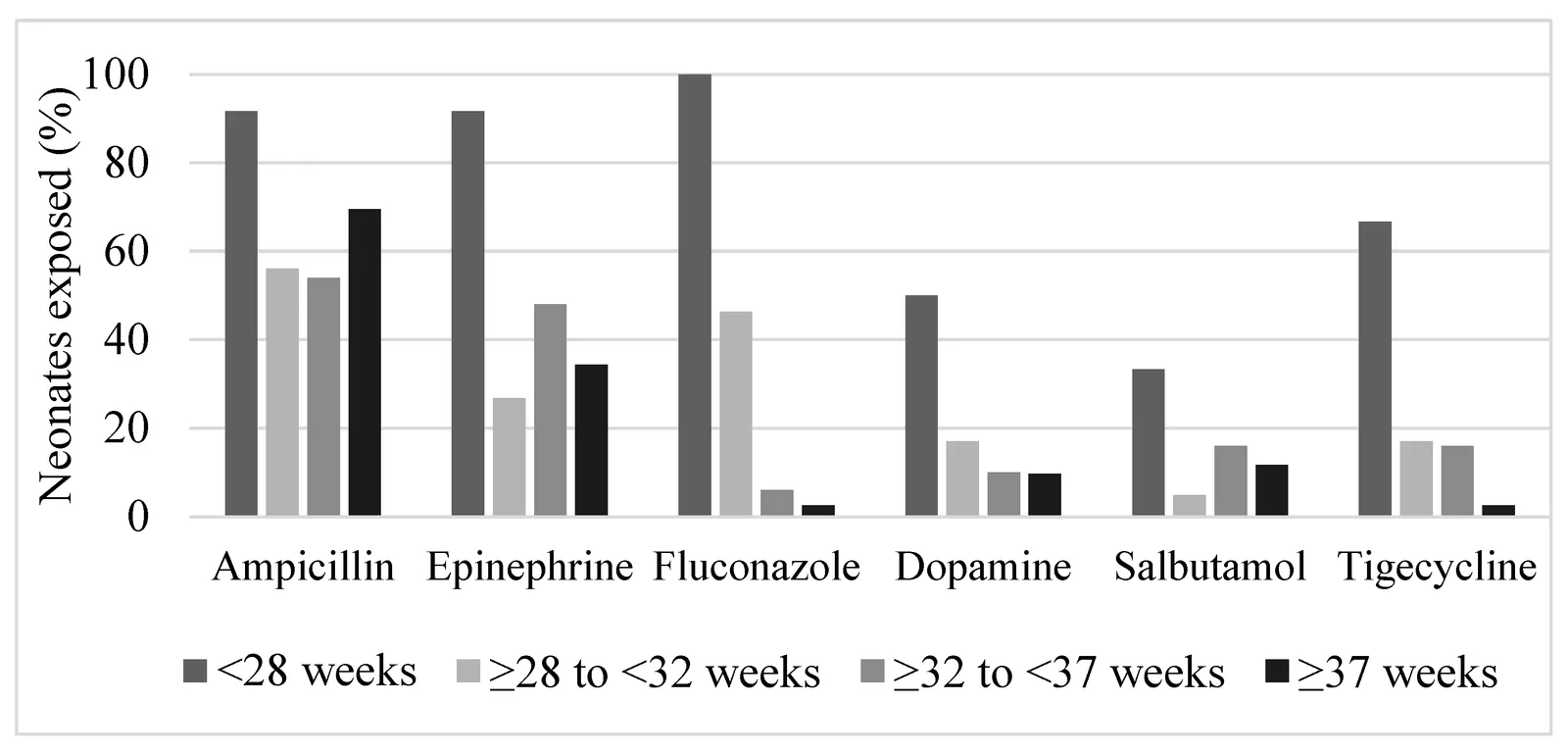
Patient exposure to selected off-label medications according to gestational age.

Figure 2 presents patient-level exposure to six commonly used off-label medications across gestational-age groups. Descriptive variation in exposure was observed across the four groups, with the <28-week group showing the highest proportion of exposed neonates for each of the six medications presented. These comparisons are descriptive and should not be interpreted as evidence of statistically significant between-group differences.

Bars represent the percentage of neonates within each gestational-age group who received the respective medication at least once during hospitalization. The figure is presented descriptively; no pairwise statistical comparisons are indicated. The gestational-age group denominators were 12, 41, 50, and 154 neonates, respectively.

## 4. Discussion

To the best of our knowledge, this study is the first to retrospectively assess the regulatory labeling status of medications administered to critically ill neonates in Serbia over a one-year period. Based on 1919 medication prescription records, our findings demonstrate extensive medication utilization in this population. However, for many medications used in neonatal care, regulatory information specific to this vulnerable population remains limited.

The proportion of prescription records classified as off-label in our study was 85.6% (1642/1919). This was higher than the rates reported in several studies conducted globally, where OL medication use ranged from 23% to 70% [5,14,15,16,17,18,19,20]. These variations may be explained by differences in the availability of medications approved for neonatal use, study design, sample size and population characteristics, and national pediatric prescribing policies, as well as the methodological approaches and definitions of OL use employed across studies [8,17]. The methodological approach used to define off-label prescribing is a critical determinant of reported prevalence and likely contributes substantially to the variability observed among otherwise comparable studies. A recent systematic review highlighted the lack of a uniform definition across countries, with most studies applying national regulatory criteria, thereby limiting direct comparisons [8]. For instance, while studies from Southern Italy and Ireland identified caffeine as the most frequently used OL medication in neonates [15,21], in our study it was considered on-label according to the national SmPC.

An additional consideration relates to the regulatory definition of off-label prescribing adopted in the present study. Several medications, particularly antibiotics such as gentamicin and ampicillin, were classified as off-label because the prescribed neonatal dose or dosing interval differed from the approved SmPC, despite these regimens being widely recommended in contemporary neonatal guidelines and routinely used in clinical practice. Consequently, the use of regulatory SmPC criteria may overestimate the prevalence of off-label prescribing when compared with classifications based on current evidence-based neonatal practice. Nevertheless, the use of nationally approved SmPCs ensured a standardized and reproducible classification approach and allowed direct assessment of prescribing in relation to the existing regulatory framework in Serbia.

At the patient level, 252/257 neonates (98.1%) were exposed to at least one off-label medication during hospitalization. This rate is comparable to, but slightly higher than, those reported in previous studies, which documented exposure rates ranging from 94.8% to 99.5% [5,16,22,23]. However, the exposure rates reported in Germany, Ireland, and Iran—ranging from 69.7% to 89.9% [14,15,24]—are lower than in our study, yet they still reflect a high prevalence of OL medication use [14,15,24]. Such widespread OL prescribing likely reflects the scarcity of safety and efficacy data for neonatal populations in premarketing trials, compelling clinicians to rely on OL options when approved alternatives are unavailable.

At the therapeutic-class level, anti-infectives for systemic use accounted for the largest number of prescription records, followed by nervous system and cardiovascular medications. This general pattern is consistent with previous neonatal studies in which systemic anti-infectives were among the most frequently used therapeutic classes [8]. Although the most common diagnoses in our cohort were respiratory distress syndrome, preterm birth with birth weight between 1000 and 2499 g, and moderate to mild birth asphyxia—conditions not primarily infectious in nature—the frequent and prolonged use of antibiotics may reflect routine empirical treatment to prevent or manage suspected early-onset neonatal sepsis. This practice is common in NICUs due to the high vulnerability of preterm infants to infections, the nonspecific clinical presentation of neonatal sepsis, and the need for rapid intervention [25].

Among antibiotics, gentamicin, ampicillin, and ceftazidime had the highest patient exposure rates among the off-label agents evaluated. This finding aligns with data from studies conducted in Spain and Iran [14,26] and is consistent with findings from India, which reported ampicillin, followed by vancomycin and ceftazidime, as the most commonly prescribed OL antibiotics [17]. Furthermore, a comprehensive review of 23 studies across 15 countries and four continents supports these trends: ampicillin was cited in 8 studies, and gentamicin in 6 [8]. A slight variation is noted in a study from Iran, where ceftriaxone and gentamicin were reported as the most and least frequently used antibiotics, respectively [14]. Despite slight differences, antibiotics remain the most commonly prescribed medication class in NICUs, with variations across units influenced by clinical protocols, local resistance patterns, and medication availability [27,28,29]. Notably, ampicillin and gentamicin continue to represent the global standard of care for the empirical treatment of early-onset neonatal sepsis—a leading and potentially life-threatening condition in neonates, particularly among preterm infants [8]. These findings emphasize the persistent reliance on off-label antibiotic use in NICUs, reflecting both clinical necessity and the limited availability of approved neonatal medications. They further support the development of local and national evidence-based guidelines for the diagnosis and management of early-onset neonatal sepsis, as well as standardized dosing recommendations for the most frequently used off-label antibiotics in neonatal practice.

In the present study, the most common reasons for OL prescribing were related to patient age and dose or dosing interval. These findings align with a recent systematic review [8], which identified the use of medications outside the approved age range as the most frequently reported reason for OL use—cited as the primary cause in nine studies, while prescribing outside the authorized dose range was also reported as the main justification in another nine studies. Our findings are also consistent with an Indian study, which reported that anti-infectives for systemic use (group J) were most frequently prescribed OL with respect to dose, followed by frequency and age, while nervous system medications (ATC group N), particularly anticonvulsants and sedatives, were predominantly used OL due to age-related restrictions [30]. These consistent patterns across studies underscore the persistent gap between regulatory approvals and clinical practice in neonatal pharmacotherapy. The frequent OL use of medications due to age and dosing limitations highlights the urgent need for more age-appropriate formulations, dosing guidelines, and clinical trials specifically designed for the neonatal population. Without such evidence, clinicians are often required to rely on empirical prescribing practices. Although the present study did not evaluate clinical outcomes, medication errors, or adverse drug reactions, these findings highlight the importance of generating high-quality evidence to support neonatal prescribing and should encourage future prospective studies addressing these clinically relevant outcomes.

Medication utilization patterns varied across gestational-age groups; however, these differences should be interpreted in the context of substantial differences in hospitalization duration. Previous studies have similarly reported a high prevalence of off-label medication use among preterm neonates [15,23,31]. Extremely preterm neonates had the longest hospital stays, with a median of 32.5 days, compared with 12.0, 8.0, and 7.0 days in the ≥28 to <32, ≥32 to <37, and ≥37 weeks groups, respectively. Thus, their cumulative medication exposure during hospitalization may partly reflect a longer period of opportunity for pharmacological treatment in addition to their greater clinical severity and therapeutic needs. Importantly, when medication exposure was descriptively standardized for hospitalization duration, the median number of distinct medications per patient-day was 0.45, 0.58, 0.86, and 0.83 across the four gestational-age groups, respectively. Therefore, the previously observed pattern of greater cumulative medication exposure among less mature neonates did not translate into a corresponding gradient after accounting descriptively for exposure time. These findings should consequently be interpreted as unadjusted associations rather than evidence of an independent effect of gestational age on medication exposure. Although extremely preterm neonates represent a clinically vulnerable population with substantial therapeutic requirements, further studies incorporating measures of illness severity and multivariable adjustment are needed to determine the independent contribution of gestational age to medication utilization.

Patient-level off-label medication exposure was high in our study, with 252/257 neonates (98.1%) exposed to at least one off-label medication, whereas exposure to unlicensed medications was relatively uncommon, occurring in 26/257 neonates (10.1%). This patient-level unlicensed exposure was lower than estimates reported in several previous studies, in which unlicensed use ranged from 1.9% to 56% [14,15,16,32]. These variations may be attributed to the fact that some countries rely more heavily on hospital formularies or magistral preparations, which may be considered UL depending on local definitions. Such regulatory heterogeneity can significantly impact how medications are classified and reported across studies.

Beyond describing prescribing frequencies, the present study provides one of the first comprehensive regulatory assessments of off-label and unlicensed medication use in a Serbian neonatal intensive care setting. By systematically classifying all prescribed medications according to the nationally approved SmPC, this study highlights the considerable gap between regulatory authorization and routine neonatal clinical practice. These findings not only provide baseline data for Serbia but also contribute to the growing international literature demonstrating that widespread off-label prescribing in NICUs largely reflects the limited availability of medications specifically authorized for neonatal use rather than inappropriate prescribing practices. Consequently, our results may support future regulatory initiatives, national prescribing guidelines, and multicenter studies aimed at optimizing neonatal pharmacotherapy.

### Strengths and Limitations

The present study has several methodological strengths. It represents the first comprehensive assessment of off-label medication use in Serbian neonatal intensive care practice; however, its principal strengths extend beyond its novelty. All medications were systematically evaluated according to the approved SmPC authorized in the Republic of Serbia, ensuring a standardized and reproducible classification of OL and UL prescribing. In addition, the study was based on detailed patient-level medication data obtained from a tertiary neonatal intensive care unit, allowing comprehensive characterization of prescribing practices in critically ill neonates. Furthermore, the stratified analysis according to gestational age and the evaluation of the underlying reasons for OL classification (e.g., age, indication, dose, or dosing interval) provided a detailed insight into prescribing patterns across clinically relevant neonatal subgroups.

Nevertheless, several limitations should be considered when interpreting the findings. First, the study was conducted in a single tertiary neonatal intensive care unit, and prescribing practices, medication availability, and clinical protocols may vary across other institutions and regions, potentially limiting the generalizability of the results. Furthermore, eight neonates who died within the first 24 h after admission were excluded from the analysis, whereas 13 neonates who died after more than 24 h of hospitalization remained included in the study cohort and contributed to all analyses. Consequently, the present findings primarily reflect medication utilization among neonates surviving beyond the first 24 h of neonatal intensive care unit hospitalization. The exclusion of neonates who died within the first 24 h may have resulted in underrepresentation of medication exposure among the most critically ill neonates during the earliest phase of intensive care and may therefore have introduced a degree of selection bias. Accordingly, the findings should not be extrapolated to neonates with very early mortality. Future studies including this subgroup may provide additional insight into medication exposure during the earliest phase of neonatal intensive care. In addition, the lack of systematic follow-up data precluded evaluation of short- and long-term clinical outcomes associated with off-label medication exposure. Consequently, the present study should be interpreted as a descriptive pharmacoepidemiological analysis of prescribing patterns rather than an assessment of the clinical effectiveness or safety of off-label medication use. Finally, comparisons of medication utilization across gestational-age groups were not adjusted for clinical severity or other potential confounders. Although hospitalization duration differed substantially across gestational-age groups and medication exposure was additionally expressed as distinct medications per patient-day, this descriptive standardization does not replace multivariable adjustment. Therefore, the observed differences across gestational-age groups should not be interpreted as demonstrating an independent effect of gestational age on medication exposure.

## 5. Conclusions

This study demonstrates a high prevalence of off-label prescribing in a Serbian tertiary neonatal intensive care unit, whereas patient exposure to unlicensed medications was relatively uncommon. Anti-infectives for systemic use represented the predominant therapeutic class, while age and dosing discrepancies were among the principal reasons for off-label classification. Medication utilization varied across gestational-age groups, but these differences should be interpreted in the context of substantial differences in hospitalization duration and potential differences in clinical severity and do not demonstrate an independent effect of gestational age. The findings characterize current prescribing patterns and regulatory gaps in neonatal pharmacotherapy and identify priorities for future pharmacokinetic, effectiveness, and safety research, including studies accounting for illness severity and other potential confounders.

## Figures and Tables

**Table 1:** Demographic and clinical characteristics of neonates admitted to the neonatal intensive care unit.

Variable	*n* (%)
Sex (*n*%)	
Male	151 (58.8)
Female	106 (41.2)
Gestational age, mean ± standard deviation, weeks	35.6 ± 4.2
<28 weeks (*n* = 12)	12 (4.7)
≥28 to <32 weeks (*n* = 41)	41 (16.0)
≥32 to <37 weeks (*n* = 50)	50 (19.4)
≥37 weeks (*n* = 154)	154 (59.9)
Documented diagnoses	
P22.0 Respiratory distress syndrome of newborn	113 (44.0)
P21.0 Severe perinatal asphyxia	41 (16.0)
P21.1 Mild and moderate perinatal asphyxia	79 (30.7)
P36.9 Neonatal sepsis, unspecified	30 (11.7)
P91.6 Neonatal hypoxic–ischemic encephalopathy	29 (11.3)
P07.1 Other low birth weight newborn (birth weight 1000–2499 g)	75 (29.2)
Other	138 (53.7)
Birth weight (g), (mean ± standard deviation)	2589.6 ± 1028.9
Birth length (cm), (mean ± standard deviation)	46.1 ± 6.5
Length of hospitalization (*n* = 257), median (interquartile range), days	8 (5–11)
<28 weeks (*n* = 12)	32.5 (17.8–50.3)
≥28 to <32 weeks (*n* = 41)	12.0 (8.0–20.0)
≥32 to <37 weeks (*n* = 50)	8.0 (5.3–13.5)
≥37 weeks (*n* = 154)	7.0 (5.0–8.8)
Distinct medications per patient-day, median (interquartile range)	
<28 weeks (*n* = 12)	0.45 (0.40–0.63)
≥28 to <32 weeks (*n* = 41)	0.58 (0.47–0.73)
≥32 to <37 weeks (*n* = 50)	0.86 (0.65–1.00)
≥37 weeks (*n* = 154)	0.83 (0.60–1.14)
Apgar score (1st min) (mean ± standard deviation)	6.4 ± 2.7
Apgar score (5th min) (mean ± standard deviation)	7.7 ± 2.0

Length of hospitalization and the number of distinct medications per patient-day are presented as median (interquartile range). Distinct medications per patient-day were calculated as the number of distinct study-included medications received by each neonate divided by the duration of hospitalization in days. Patients could have more than one documented diagnosis and could therefore contribute to more than one diagnostic category; consequently, percentages for documented diagnoses do not sum to 100%. The “Other” category includes patients with one or more documented diagnoses other than the six diagnoses presented separately in the table.

**Table 2:** Medication exposure according to Anatomical Therapeutic Chemical groups by gestational age.

Anatomical Therapeutic Chemical Group	<28 Weeks: Prescriptions; Exposed Neonates, *n*/*N* (%)	≥28 to <32 Weeks: Prescriptions; Exposed Neonates, *n*/*N* (%)	≥32 to <37 Weeks: Prescriptions; Exposed Neonates, *n*/*N* (%)	≥37 Weeks: Prescriptions; Exposed Neonates, *n*/*N* (%)	Total: Prescriptions; Exposed Neonates, *n*/*N* (%)
Alimentary tract and metabolism	6 5/12 (41.7%)	11 10/41 (24.4%)	8 8/50 (16.0%)	19 18/154 (11.7%)	44 41/257 (16.0%)
Blood and blood-forming organs	1 1/12 (8.3%)	5 3/41 (7.3%)	3 3/50 (6.0%)	8 8/154 (5.2%)	17 15/257 (5.8%)
Cardiovascular system	29 9/12 (75.0%)	35 16/41 (39.0%)	64 32/50 (64.0%)	178 93/154 (60.4%)	306 150/257 (58.4%)
Dermatologicals	2 2/12 (16.7%)	4 4/41 (9.8%)	2 2/50 (4.0%)	5 5/154 (3.2%)	13 13/257 (5.1%)
Genitourinary system and sex hormones	2 2/12 (16.7%)	2 2/41 (4.9%)	0 0/50 (0.0%)	1 1/154 (0.6%)	5 5/257 (1.9%)
Systemic hormonal preparations, excluding sex hormones and insulins	6 5/12 (41.7%)	5 5/41 (12.2%)	3 3/50 (6.0%)	18 17/154 (11.0%)	32 30/257 (11.7%)
Anti-infectives for systemic use	101 12/12 (100.0%)	182 41/41 (100.0%)	178 50/50 (100.0%)	455 147/154 (95.5%)	916 250/257 (97.3%)
Antineoplastic and immunomodulating agents	0 0/12 (0.0%)	1 1/41 (2.4%)	0 0/50 (0.0%)	0 0/154 (0.0%)	1 1/257 (0.4%)
Musculoskeletal system	3 3/12 (25.0%)	11 11/41 (26.8%)	14 14/50 (28.0%)	57 57/154 (37.0%)	85 85/257 (33.1%)
Nervous system	32 12/12 (100.0%)	64 41/41 (100.0%)	69 37/50 (74.0%)	177 108/154 (70.1%)	342 198/257 (77.0%)
Respiratory system	19 10/12 (83.3%)	28 22/41 (53.7%)	31 21/50 (42.0%)	74 51/154 (33.1%)	152 104/257 (40.5%)
Sensory organs	2 2/12 (16.7%)	0 0/41 (0.0%)	1 1/50 (2.0%)	2 2/154 (1.3%)	5 5/257 (1.9%)
Various	0 0/12 (0.0%)	0 0/41 (0.0%)	0 0/50 (0.0%)	1 1/154 (0.6%)	1 1/257 (0.4%)

Prescription count represents the number of distinct medications prescribed to neonates within each therapeutic class; repeated doses or administrations of the same medication in the same patient were not counted as separate prescriptions. A single neonate could contribute more than one prescription within the same therapeutic class if more than one distinct medication from that class was prescribed. Patient exposure represents the number and proportion of neonates who received at least one medication from the respective therapeutic class.

**Table 3:** Patient exposure to the most frequently prescribed off-label and unlicensed medications according to gestational age.

Medication	Route of Administration	Gestational Age < 28 Weeks, *n*/*N* (%)	Gestational Age ≥ 28 to <32 Weeks, *n*/*N* (%)	Gestational Age ≥ 32 to <37 Weeks, *n*/*N* (%)	Gestational Age ≥ 37 Weeks, *n*/*N* (%)	Overall Patient Exposure, *n*/*N* (%)	Reason for Off-Label or Unlicensed Classification
Gentamicin	Intravenous	12/12 (100.0%)	40/41 (97.6%)	46/50 (92.0%)	135/154 (87.7%)	233/257 (90.7%)	Dose/dosing interval
Ampicillin	Intravenous	11/12 (91.7%)	23/41 (56.1%)	27/50 (54.0%)	107/154 (69.5%)	168/257 (65.4%)	Dose/dosing interval
Ceftazidime	Intravenous	11/12 (91.7%)	12/41 (29.3%)	22/50 (44.0%)	96/154 (62.3%)	141/257 (54.9%)	Dose/dosing interval
Fentanyl	Continuous intravenous infusion	8/12 (66.7%)	11/41 (26.8%)	20/50 (40.0%)	73/154 (47.4%)	112/257 (43.6%)	Dose
Furosemide	Intravenous	7/12 (58.3%)	9/41 (22.0%)	23/50 (46.0%)	67/154 (43.5%)	106/257 (41.2%)	Age
Allopurinol	Enteral (via gastric tube)	3/12 (25.0%)	11/41 (26.8%)	14/50 (28.0%)	57/154 (37.0%)	85/257 (33.1%)	Age
Benzylpenicillin	Intravenous	1/12 (8.3%)	18/41 (43.9%)	21/50 (42.0%)	34/154 (22.1%)	74/257 (28.8%)	Age
Meropenem	Intravenous	11/12 (91.7%)	19/41 (46.3%)	16/50 (32.0%)	19/154 (12.3%)	65/257 (25.3%)	Dose/dosing interval
Midazolam	Intravenous	4/12 (33.3%)	4/41 (9.8%)	12/50 (24.0%)	43/154 (27.9%)	63/257 (24.5%)	Age
Budesonide	Inhalation	11/12 (91.7%)	9/41 (22.0%)	13/50 (26.0%)	30/154 (19.5%)	63/257 (24.5%)	Age
Dobutamine	Continuous intravenous infusion	6/12 (50.0%)	5/41 (12.2%)	6/50 (12.0%)	34/154 (22.1%)	51/257 (19.8%)	Age
Fluconazole	Intravenous	12/12 (100.0%)	19/41 (46.3%)	3/50 (6.0%)	4/154 (2.6%)	38/257 (14.8%)	Age
Acetaminophen	Intravenous	4/12 (33.3%)	7/41 (17.1%)	10/50 (20.0%)	16/154 (10.4%)	37/257 (14.4%)	Age
Colistimethate sodium	Intravenous	9/12 (75.0%)	12/41 (29.3%)	6/50 (12.0%)	8/154 (5.2%)	35/257 (13.6%)	Dose/dosing interval
Phenobarbital	Intravenous	5/12 (41.7%)	2/41 (4.9%)	8/50 (16.0%)	19/154 (12.3%)	34/257 (13.2%)	Age
Dopamine	Continuous intravenous infusion	6/12 (50.0%)	7/41 (17.1%)	5/50 (10.0%)	15/154 (9.7%)	33/257 (12.8%)	Age
Salbutamol	Inhalation	4/12 (33.3%)	2/41 (4.9%)	8/50 (16.0%)	18/154 (11.7%)	32/257 (12.5%)	Age
Tigecycline	Intravenous	8/12 (66.7%)	7/41 (17.1%)	8/50 (16.0%)	4/154 (2.6%)	27/257 (10.5%)	Age
Pantoprazole	Intravenous	0/12 (0.0%)	6/41 (14.6%)	5/50 (10.0%)	11/154 (7.1%)	22/257 (8.6%)	Age
Linezolid	Intravenous	4/12 (33.3%)	3/41 (7.3%)	4/50 (8.0%)	4/154 (2.6%)	15/257 (5.8%)	Age
Insulin	Continuous intravenous infusion	4/12 (33.3%)	2/41 (4.9%)	1/50 (2.0%)	7/154 (4.5%)	14/257 (5.4%)	Age
Imipenem	Intravenous	2/12 (16.7%)	2/41 (4.9%)	2/50 (4.0%)	4/154 (2.6%)	10/257 (3.9%)	Age
Dexamethasone	Intravenous	0/12 (0.0%)	2/41 (4.9%)	0/50 (0.0%)	7/154 (4.5%)	9/257 (3.5%)	Age
Fraxiparine	Subcutaneous	1/12 (8.3%)	1/41 (2.4%)	0/50 (0.0%)	5/154 (3.2%)	7/257 (2.7%)	Age
Heparin	Continuous intravenous infusion	0/12 (0.0%)	2/41 (4.9%)	3/50 (6.0%)	2/154 (1.3%)	7/257 (2.7%)	Age
Metronidazole	Intravenous	1/12 (8.3%)	1/41 (2.4%)	3/50 (6.0%)	1/154 (0.6%)	6/257 (2.3%)	Age
Acyclovir	Intravenous	0/12 (0.0%)	0/41 (0.0%)	0/50 (0.0%)	4/154 (2.6%)	4/257 (1.6%)	Age
Nystatin	Topical	1/12 (8.3%)	2/41 (4.9%)	0/50 (0.0%)	1/154 (0.6%)	4/257 (1.6%)	Age
Alprostadil	Continuous intravenous infusion	0/12 (0.0%)	1/41 (2.4%)	1/50 (2.0%)	2/154 (1.3%)	4/257 (1.6%)	Age
Adenosine	Intravenous	0/12 (0.0%)	0/41 (0.0%)	1/50 (2.0%)	2/154 (1.3%)	3/257 (1.2%)	Age
Epinephrine	Intravenous	11/12 (91.7%)	11/41 (26.8%)	24/50 (48.0%)	53/154 (34.4%)	99/257 (38.5%)	Age
Levofloxacin	Intravenous	1/12 (8.3%)	1/41 (2.4%)	1/50 (2.0%)	0/154 (0.0%)	3/257 (1.2%)	Age
Other (24 medications)	Various	6/12 (50.0%)	8/41 (19.5%)	3/50 (6.0%)	14/154 (9.1%)	31/257 (12.1%)	Various
Povidone-iodine	Ophthalmic	2/12 (16.7%)	4/41 (9.8%)	2/50 (4.0%)	4/154 (2.6%)	12/257 (4.7%)	Modified formulation
Propranolol	Enteral (via gastric tube)	0/12 (0.0%)	1/41 (2.4%)	1/50 (2.0%)	4/154 (2.6%)	6/257 (2.3%)	Modified formulation
Ursodeoxycholic acid	Enteral (via gastric tube)	2/12 (16.7%)	2/41 (4.9%)	1/50 (2.0%)	1/154 (0.6%)	6/257 (2.3%)	Modified formulation
Captopril	Enteral (via gastric tube)	1/12 (8.3%)	0/41 (0.0%)	3/50 (6.0%)	0/154 (0.0%)	4/257 (1.6%)	Modified formulation

Patient exposure is presented as *n*/*N* (%), where *n* is the number of neonates who received the corresponding medication at least once during hospitalization, and *N* is the total number of neonates in the respective gestational-age group. Group denominators were 12 neonates at <28 weeks, 41 at ≥28 to <32 weeks, 50 at ≥32 to <37 weeks, and 154 at ≥37 weeks; the overall denominator was 257 neonates. A neonate was counted only once for each medication, irrespective of repeated doses or administrations. “Other” comprises 24 less frequently prescribed off-label medications. The table is descriptive; no pairwise statistical comparisons between gestational-age groups are presented.

## Data Availability

The data used and analysed during the current study are available from the corresponding author on reasonable request.

## Appendix A

**Table A1:** Patient exposure to on-label medications according to gestational age.

Medication	Route of Administration	Gestational Age < 28 Weeks, *n*/*N* (%)	Gestational Age ≥ 28 to <32 Weeks, *n*/*N* (%)	Gestational Age ≥ 32 to <37 Weeks, *n*/*N* (%)	Gestational Age ≥ 37 Weeks, *n*/*N* (%)	Overall Patient Exposure, *n*/*N* (%)
Caffeine citrate	Intravenous	12/12 (100.0%)	39/41 (95.1%)	16/50 (32.0%)	13/154 (8.4%)	80/257 (31.1%)
Vancomycin	Intravenous	9/12 (75.0%)	15/41 (36.6%)	11/50 (22.0%)	36/154 (23.4%)	71/257 (27.6%)
Poractant alfa	Endotracheal instillation	5/12 (41.7%)	17/41 (41.5%)	10/50 (20.0%)	24/154 (15.6%)	56/257 (21.8%)
Amikacin	Intravenous	6/12 (50.0%)	2/41 (4.9%)	3/50 (6.0%)	1/154 (0.6%)	12/257 (4.7%)
Ampicillin/sulbactam	Intravenous	4/12 (33.3%)	3/41 (7.3%)	2/50 (4.0%)	0/154 (0.0%)	9/257 (3.5%)
Hydrocortisone	Intravenous	1/12 (8.3%)	1/41 (2.4%)	0/50 (0.0%)	5/154 (3.2%)	7/257 (2.7%)
Ibuprofen	Intravenous	2/12 (16.7%)	2/41 (4.9%)	0/50 (0.0%)	1/154 (0.6%)	5/257 (1.9%)
Dexamethasone/neomycin	Eye drops	1/12 (8.3%)	0/41 (0.0%)	1/50 (2.0%)	2/154 (1.3%)	4/257 (1.6%)

Patient exposure is presented as *n*/*N* (%), where n is the number of neonates who received the corresponding medication at least once during hospitalization, and *N* is the total number of neonates in the respective gestational-age group. Group denominators were 12 neonates at <28 weeks, 41 at ≥28 to <32 weeks, 50 at ≥32 to <37 weeks, and 154 at ≥37 weeks; the overall denominator was 257 neonates. A neonate was counted only once for each medication irrespective of repeated doses or administrations.

**Table A2:** Number of distinct off-label medications per neonate according to gestational age.

Gestational Age	*n*	Distinct Off-Label Medications Per Neonate, Median (Interquartile Range)
<28 weeks	12	10.5 (8.5–17.0)
≥28–<32 weeks	41	5.0 (3.0–7.0)
≥32–<37 weeks	50	6.0 (3.0–8.0)
≥37 weeks	154	5.0 (3.0–8.0)

Values are presented as median (interquartile range). The overall comparison across gestational-age groups was performed using the Kruskal–Wallis test (H = 18.29, *p* = 0.00038, ε^2^ = 0.060). No post hoc pairwise comparisons were performed.
